# A Community of Curious Souls: An Analysis of Commenting Behavior on TED Talks Videos

**DOI:** 10.1371/journal.pone.0093609

**Published:** 2014-04-09

**Authors:** Andrew Tsou, Mike Thelwall, Philippe Mongeon, Cassidy R. Sugimoto

**Affiliations:** 1 School of Informatics and Computing, Indiana University, Bloomington, Indiana, United States of America; 2 School of Technology, University of Wolverhampton, Wolverhampton, United Kingdom; 3 École de bibliothéconomie et des sciences de l’information, Université de Montréal, Montréal, Québec, Canada; University of Warwick, United Kingdom

## Abstract

The TED (Technology, Entertainment, Design) Talks website hosts video recordings of various experts, celebrities, academics, and others who discuss their topics of expertise. Funded by advertising and members but provided free online, TED Talks have been viewed over a billion times and are a science communication phenomenon. Although the organization has been derided for its populist slant and emphasis on entertainment value, no previous research has assessed audience reactions in order to determine the degree to which presenter characteristics and platform affect the reception of a video. This article addresses this issue via a content analysis of comments left on both the TED website and the YouTube platform (on which TED Talks videos are also posted). It was found that commenters were more likely to discuss the characteristics of a presenter on YouTube, whereas commenters tended to engage with the talk content on the TED website. In addition, people tended to be more *emotional* when the speaker was a woman (by leaving comments that were either positive or negative). The results can inform future efforts to popularize science amongst the public, as well as to provide insights for those looking to disseminate information via Internet videos.

## Introduction

Disseminating knowledge is a key component of scientific scholarship, for without sharing one’s findings, there is little point in doing research. The manner in which science is communicated is therefore of tremendous importance, and is rife with potential pitfalls. There is evidence that scientists are not formally trained as communicators [1], and it would not be surprising if an individual supremely gifted in mathematics (for example) would lack the verbal communication skills that might be expected of a linguist. The myriad complications that haunt human communication are evidenced in scholarly activity by the fact that the “diversity of communication outlets and specialized terminologies makes it hard for many non-specialists (and even specialists) to locate important studies” [2]. But science communication is not solely about disseminating information to an elite group of individuals, and locating works that discuss key concepts or breakthroughs should not be an arduous undertaking. It would make sense, then, that popularization of science is an issue that should be at the forefront of scholarly communication, although this is not necessarily the case. For example, Davies optimistically suggested that “scientists and engineers are at the very least aware of a push toward public communication, and in many cases have taken part in one or more science communication activities…scientists and engineers today have the funds, the opportunities, and often the desire for public engagement” [3]. Some academic institutions have enlisted professionals to aid researchers in the act of public dissemination [4], but some commentators are not quite so sanguine about the situation. It has been found that “only a minority of scientists regularly engage” in popularization efforts [5], and many scientists also consider popularization to be an activity that falls outside the scope of their job duties [6], [7]. All the same, communicating scientific knowledge to the public is frequently perceived as an integral part of scholarly communication.

The Internet has made possible a variety of communication approaches, given that it welds “the information richness of print with the demonstration power of broadcast in a seamless, accessible, interactive fashion” [8]. The National Science Board has reported that the Internet is “the main source of information for learning about specific scientific issues” [9], and there is evidence that YouTube videos relating to science and technology tend to receive heavy discussion relative to other categories of videos [10]. In terms of scientific communication facilitated by the Internet, disseminators face two primary problems: competition with non-scientific sources, and audiences that can range from unreceptive to actively destructive. While the latter has always been an issue for public speakers or communicators, the nature of online discourse makes for an environment that poses unique challenges to scientists. Brossard and Scheufele found that “the medium can have a surprisingly potent effect on the message. Comments from some readers…can significantly distort what other readers think was reported in the first place” [11], and these comments are often motivated by the fact that it is currently “socially acceptable, to deny scientific fact” [12]. There is evidence that “online newspaper articles are not consumed in isolated fashion as they used to be and are now contextualized by readers’ comments” [13], and some news websites have responded to the potentially deleterious nature of user comments by simply disabling their comments feature altogether [14].

One of the most successful outreach initiatives in the digital age is the TED website, which primarily hosts videos of presentations given at TED conferences by academics, industry figures, artists, musicians, and a variety of other individuals. These videos have been viewed over a billion times on the TED website [15] in addition to hundreds of millions of views on YouTube [16], which seems to be more than any other science communication initiative. The TED conference theoretically focuses on Technology, Entertainment, and Design, but TED is frequently perceived as a venue for those avenues of research that are considered “important” (primarily in the hard sciences). As of November 4, 2013, there were 520 “technology” talks available on the TED website, 265 “entertainment” talks, 313 “design” talks, and 397 “science” talks. Although there is a degree of overlap between the categories, it is interesting to note that “science” is a more frequent topic than two of the subjects that give TED its name. Other common subjects include “global issues” (375), “business” (252), and even “politics” (132). Clearly, then, TED has evolved into a platform for discussing weighty topics, including issues of scientific concern. This is particularly important in light of the relative dearth of “popular” science communication when compared to mainstream texts, videos, and speeches pertaining to the humanities and social sciences [17]. TED’s slogan is simply “Ideas worth spreading,” which implies a broad focus that extends to include all topics of potential interest to a wide audience.

TED Talks have attracted criticism for a variety of reasons. There is a significant gender bias in relation to the videos that are posted on the TED site, as only 27% of these talks are presentations by females [18], and various blogs and mass media sources have commented unfavorably about the populist and entertainment-heavy nature of TED videos, suggesting that TED Talks are not so much critical assessments of relevant topics as they are enthusiastic sales pitches [19]–[22]. One would presume that the types of scientists who are willing to speak at TED are those that are adept at simplifying their work and entertaining a lay public, which tends to favor “rock star” scientists over those whose research may perhaps be more innovative or profound. TED, then, falls somewhere on a spectrum bookended by “entertainment” and “education,” and determining just where it falls on this spectrum (at least as measured by audience reaction) is a focal point of this study.

Whereas TED maintains a reputation as something of an intellectual fount (at least within the context of the Internet’s non-academic sphere), the YouTube site is decidedly less revered by a scholarly elite. Instead, it is one of the most populist websites extant. YouTube is “the most popular user generated content” website on the Web [23], ranks as the third most popular website in the world [24], and has been used to varying degrees of success for a variety of pedagogical activities within the classroom [25]–[31]. In addition, medical information posted on YouTube has been used by the indigent in order to obtain health care that would not otherwise be available [32]. Nevertheless, despite the site’s popularity, it remains to be discovered just how deeply viewers engage with the material posted on YouTube, particularly in regard to videos that are intended to be or tagged as educational. In addition, research is required to investigate the characteristics of individuals who seek out science videos on their own, as opposed to gaining exposure to these videos via formal educational establishments.

The TED Talks website states that “we believe passionately in the power of ideas to change attitudes, lives and, ultimately, the world. So we’re building a clearinghouse of free knowledge from the world’s most inspired thinkers, and also a community of curious souls to engage with ideas and each other” [33]. Accordingly, our study attempts to discover just how deeply viewers are engaging with the ideas presented in TED videos, as well as to determine how these viewers are interacting with each other. This is measured by analyzing the content and sentiment of comments left on either the TED website or on the corresponding YouTube page (all talks that are posted on the TED website are also posted to the TED director’s YouTube channel). A number of variables are considered, including platform (i.e., the TED website or YouTube) and the characteristics of each presenter (i.e., academic status and gender). By analyzing commenters’ behavior on YouTube and the TED Talks website, we can gain insight into the degree to which viewers engage with speakers, talk content (i.e., ideas), and other commenters. Specifically, we seek to answer the following research questions:

1. **Is there a significant difference in the type of comments according to platform?**
2. **Are significant differences in commenting observed according to presenter characteristics?**

Although previous research has investigated the characteristics of TED Talks presenters [18] and the popularity of TED videos as measured by YouTube “likes” [16], the manner in which people *engage* with these talks has yet to be investigated. Given the popularity of TED Talks and the high visibility that a TED Talk can endow upon a presenter or an idea, there is a need for a more robust understanding of the community that is associated with these videos. For example, it has been shown that women are underrepresented on the TED Talks website, in the sense that less than a quarter of presenters are female [18]; this study proposes to investigate whether viewers react *differently* to women, either in terms of presenter perception or engagement with the presenter’s ideas. The Internet has allowed for broader dissemination of ideas while simultaneously allowing nearly anyone to contribute to the discussion. Accordingly, it is imperative that we understand the nature of this discourse and the manner in which ideas thrive or are ignored. The results can be used to gain insight into online communication activities. In addition, scientists concerned with popularization can draw upon our results in order to plan their dissemination practices. If it is found that people are not talking about science or ideas in the comments, scientists will continue to treat TED as another mass media outlet; conversely, if it is found that people *are* discussing science (particularly on the mainstream YouTube platform), it might encourage more scientists to take advantage of modern popularization techniques.

## Methods

This project was conducted in two stages. The goal of the first stage was to identify whether commenters engaged with the topic or whether their comments were trivial (e.g., focusing on a video’s education value, interacting with other commenters without discussion of the talk, etc.). In addition, it was desired to ascertain whether the two platforms (TED and YouTube) encouraged different types of discussion. Based on the results of the first stage, the codebook was refined so as to analyze differences in commenting behavior when presenter characteristics were considered as the primary variables (stage 2). Although platform was still taken into account, the primary goal of stage 2 was to determine commenter attitudes towards talks and videos based on factors such as the presenter’s gender and academic status. In addition, whereas stage 1 was limited to videos tagged as “Science” or “Technology,” stage 2 took into consideration all videos on the TED website.

### Stage 1

#### Video sample

The raw data used for stage 1 of the study was a random collection of YouTube and TED website comments. Not all TED Talks videos are about science: some are musical or artistic performances, and others are speeches by politicians. To restrict the data to relevant videos, only those tagged in the TED website as Science or Technology were chosen, which resulted in a total of 405 videos (out of 1202).

#### Comment sample

For each of the 405 videos, up to three comments from each platform were selected at random to form a combined data set, from which training sets and a main set were extracted (all comments were selected if there were three or fewer comments for a given video). All of the comments that were analyzed (in both stages 1 and 2) have been privately archived by the authors, and will be made available upon reasonable request. It was not clear during the data collection process just how much training data would be required in order to obtain a satisfactory inter-coder agreement level (see below); accordingly, not all of the selected videos were used in the final analysis.

In the case of YouTube, Webometric Analyst was used in order to download the most recent 1,000 comments on the relevant videos. Automatic downloading of comments was not possible with the TED website. Accordingly, for each TED video: a) the number of total comments for each video was identified, b) this number was entered into a random number generator, c) three numbers were generated at random, and’ d) these numbers were used to select comments. For example, if a video had 50 comments and the random number generator produced “4,” the fourth newest comment would be selected.

#### Codebook

The categories used in the initial content analysis were developed through an integrated inductive and deductive approach. The authors approached the development of the scheme with key macro themes–i.e., differentiating between comments about the presenter, comments about the talk, and discussion with other commenters. However, the scheme was inductively expanded following independent coding of 100 random comments by members of the research team. The categories were explicitly defined, and four coders were employed to test the scheme on sets of random comments. The scheme was refined iteratively in three further stages. Each stage consisted of coders independently coding the same sets of texts and the results then compared in order to identify differences. The results were then used to refine the category descriptions and coding instructions. This process was also used to select reliable coders for this task. After the third stage, it was found that one pair of coders had acceptable levels of agreement (a Cohen’s kappa of at least 0.4) for the revised scheme’s major categories.

The objective of the classification method was to capture categories that reflected the data and related to the research questions. The categories were not mutually exclusive; accordingly, a comment could receive multiple codes. However, category 1 (comment on speaker or talk style) was made mutually exclusive with category 2 (comment on talk content), just as category 3 (interaction with previous commenter) was mutually exclusive with category 2 (in both cases, category 2 took precedence; therefore, a comment that included a discussion of talk content could *not* be coded with category 1 or category 3). This was done to capture comments that were participant interactions that did not engage with the talk content.

#### Coding

The two coders were given 600 comments made on 300 sampled TED videos selected from the combined data set. These comments were chosen from the pool of comments that were not used in any of the training sets. Five comments were removed for technical reasons (e.g., indecipherable characters), leaving a final total of 595 comments. There was one comment from YouTube and one from the TED website for each video. The comments were arranged in random order and the coders were given the comment and the title and presenter of the associated video. To avoid coder bias, the coders were not given any clues about whether each comment was taken from the TED website or from YouTube and were requested not to visit the sites in question to identify the comment or in any other way identify which site the comment came from. The coders were information science students. A short version of the coding scheme is given in Table 1. The longer descriptions included examples and reminders about similar categories that could be alternatives. For categories 1, 2 and 3, codes were assigned based on the subcategories rather the major categories.

**Table 1:** A list of the categories for the content analysis and short versions of the descriptions given to the coders.

#	Type of comment	Description
1	Comment on speaker or talk style notrelating to talk content	Praises, criticizes or makes point about speaker; Comments on presentation style. NOT comment about how good/bad the talk was.
1a	Personal anecdote (self-identification with speaker)	Describes personal experience that identifies or relates to the speaker in some way
1b	Criticism of speaker (not the talk or message)	Criticizes the speaker rather than the content of the talk; assume that any undirected criticism is directed at speaker -e.g., I hate him/her.
1c	Praise of speaker (not the talk or video)	Praises the speaker rather than the content of the talk; assume that any undirected praise is directed at speaker
1d	Comment on speaker demographics	Comments on speaker background, age, gender, appearance, etc. (also code 1a,1b,1c if relevant)
1e	Other comment on speaker	Any other comment on speaker that doesn’t fit the above categories.
1f	Comment on speaker delivery/style (with or without praise or criticism)	Comments on any aspect of the delivery of the talk or the style of the speaker (also code 1a,1b,1c if relevant). Includes comment on accent, pronunciation.
2	Comment on talk	Praises, criticizes or makes point about the content of the talk [this section is for all interactions with talk content]
2a	Personal anecdote relating to talk content	Describes in detail a personal experience that illustrates a theme in the talk or otherwise relates to the content or topic of the talk.
2b	Summarize talk or reiterate key point from talk	Gives a brief summary or overview of the talk; Quote or state a single point from the talk
2c	Praise of talk content(without any discussion of talk)	Simple statement that the talk content is good without any justification anywhere in the comment
2d	Criticism of talk content (without any discussion of talk)	Simple statement that the talk content is bad without any justification anywhere in the comment
2e	Discuss issue related to talk	Discuss a topic that is not mentioned in the talk but is topically related in some way
2f	Discuss talk - agreement/praise	Objective is to discuss something brought up in talk; commenter clearly primarily agrees with talk
2g	Discuss talk - disagreement/criticism	Objective is to discuss something brought up in talk; commenter clearly primarily disagrees with talk
2h	Discuss talk - other (without praise or criticism);	Discussion without praise, criticism, agreement or disagreement, and without contributing anything new to the argument (i.e., not 2b)
3	Other interaction with previous commenter with NO discussion of talk content	Is a reply to a previous commenter or comment WITHOUT discussion of content - ignore this section completely if there is any discussion of talk content even if the comment also includes interactions
3a	Insult previous commenter	Personal abuse directed at a previous commenter
3b	Praise previous commenter	Praise directed at a previous commenter
3c	Agree with previous comment without discussion	Do not use if any option from 2 is also selected for this comment
3d	Disagree with previous comment without discussion	Do not use if any option from 2 is also selected for this comment
3e	Any other interaction with previous commenter	Do not use if any option from 2 is also selected for this comment
4	Meta comment about TED itself	Comment about TED itself rather than just the talk
5	Spam	Irrelevant, marketing or promotional not related to talk
6	Self-promotion (related to talk)	Self-promotion of person, product or service that is directly relevant to the talk theme.
7	Other	Something in the comment that does not match any of the above categories
x	Pointer	Comment contains citation, hyperlink, book/article title or other pointer to external information

Table 2 reports the Cohen’s kappa values for the level of agreement between the two coders, broken down by each category and subcategory. A coder was said to have coded a given comment in the major categories (1, 2, and 3) if any of the associated subcategories had been selected. Any positive value for kappa indicates a level of agreement above chance, with 1 indicating perfect agreement. The Fleiss guidelines for kappa values [34] are as follows: over 0.75 is excellent, 0.40–0.75 is fair to good and below 0.40 is poor. As can be seen in table 2, all of the major categories (with the exception of the “other” category) have fair-to-good levels of agreement and are thus usable for an analysis. The major category 7 (“other”) was not analyzed.

**Table 2:** Cohen’s kappa values for each category in the scheme.

Code	Cohen’s kappa	Fleiss category
1	0.732	Fair-good
1a	0.000	Poor
1b	0.469	Fair-good
1c	0.712	Fair-good
1d	0.513	Fair-good
1e	0.100	Poor
1f	0.550	Fair-good
2	0.609	Fair-good
2a	0.567	Fair-good
2b	0.203	Poor
2c	0.589	Fair-good
2d	0.265	Poor
2e	0.311	Poor
2f	0.327	Poor
2g	0.503	Fair-good
2h	0.343	Fair-good
3	0.514	Fair-good
3a	0.657	Fair-good
3b	0.398	Poor
3c	0.291	Poor
3d	0.129	Poor
3e	0.301	Poor
4	0.655	Fair-good
5	0.422	Fair-good
6	0.665	Fair-good
7	0.297	Poor
Pointer	0.498	Fair-good

Additional coders acted as arbitrators for all cases of differences between the primary coders, and the following analysis is based upon the revised version of the codebook. The two arbitrators also checked different subsets of the results. Both were experienced and previously reliable coders. One had an information science PhD and the other was an MA English student. As a result of this arbitration, the final codes are likely to be more reliable than the Cohen’s kappa values suggest.

#### Analysis

Statistical tests were used to decide whether the proportions of videos in various categories differed between YouTube and the TED website (specifically, a differences in proportions test was used). This test assesses whether there is sufficient evidence to reject the hypothesis that two different sample proportions come from populations with the same overall (i.e., population) proportions. This test is based upon a formula taking as input the numerical difference between the two sample probabilities and the sample sizes in both cases, generating a z score that comes approximately from a normal distribution and hence can be tested against tabulated values from a standard normal distribution.

### Stage 2

Three main variables were analyzed in stage 2: 1) platform hosting the video (TED vs. YouTube); 2) gender of presenter (male vs. female); and 3) academic status (academic vs. non-academic status). A different pool of videos was used for this stage; whereas the video population in stage 1 was limited to “science and technology” videos, the sampling frame for stage 2 was constructed from the list of 1,202 videos gathered in Sugimoto and Thelwall’s earlier work on TED [16]. In a subsequent article [18], the authors coded the presenters of TED Talks into two main categories: a) male or female, and b) academic or non-academic. Accordingly, the presenter featured in each video was classified under one of four categories: female academic, female non-academic, male academic, and male non-academic. It should be noted that during the analysis conducted for this paper, it was determined that one video had been misclassified in the earlier work (one female academic had been classed as a female non-academic in Sugimoto et al. [18]. This was corrected, and thus the number of female academics in this paper (n = 49) is one higher than in the previous paper, which used the same dataset.

Stratified sampling was conducted based on the lowest common denominator–in this case, the 49 female academics. Because presenter style/appearance/etc. is an integral part of this study, unique *people* were sampled (as opposed to unique *videos*). If a person gave more than one TED talk, a random number generator was used to retain one of these talks, with the rest being discarded. In this way, 49 unique presenters were selected from each of the four categories, resulting in a total of 196 videos.

#### Comment sample

As with stage 1, Webometric Analyst was used to download relevant comments from the YouTube website, although in this stage the fifteen oldest comments were selected (as opposed to three random comments). Similarly, the fifteen oldest comments from the TED Talks website were selected, a process that was facilitated by the “Oldest first” sort feature. The TED website threads comments that are created using the “Reply” button; if these replies were clearly “newer” than other comments (based on the date stamps), they were excluded. If the situation was ambiguous (i.e., the “reply” comment and the next eligible comment had the same date stamp), the comment included in the “thread” was counted. The total number of comments sampled was 5854: 2914 comments from YouTube and 2940 comments from TED. This is less than the predicted number (30 comments multiplied by 196 videos for a total of 5880 comments), given that not every video had fifteen comments (specifically, three YouTube videos had fewer than 15 comments).

#### Codebook

Given the low kappa values obtained for the minor categories in the initial coding, the codebook was simplified for the second stage of the project, retaining the major categories (with the exception of “Spam” and “Self-promotion”) and eliminating all minor categories. A “sentiment” variable was added, requiring coders to assess each category as “positive,” “negative,” “neutral,” or “mixed.” For example, a comment that read “The presentation was nice” would be coded as “2P,” indicating that it refers to the talk content in a positive manner. Multiple codes could be assigned to a given comment, with differing sentiment codes if necessary; for example, a comment that read “You’re an idiot; her talk was great” would be coded as “2P” *and* “3N” (see table 3).

**Table 3:** Revised coding scheme for stage 2.

Type of comment		
#	Type of comment	Description
1	Comment on speaker OR talk style	Praises, criticizes or makes point about speaker; Comments on presentation style.
2	Comment on talk [this section is for all interactions with content]	Praises, criticizes or makes a point about the content of the talk
3	Interaction with previous commenter	Is a reply to a previous commenter or comment
4	Meta comment about TED itself	Comment about TED itself rather than just the talk
5	Other	Something in the comment that does not match any of the above categories; most importantly, not about the talk content or speaker in any way. DO NOT USE FOR FOREIGN COMMENTS - attempt to translate these and categorize as above. If can’t translate, mark separately
**Sentiment of comment**		
	Sentiment	
P	Positive	
N	Negative	
U	Neutral	
M	Mixed	

#### Coding

Despite this less complex coding scheme, initial attempts at coding the comments were unsatisfactory (primarily in the sentiment category). Issues such as sarcasm, ambiguous wording, Internet lingo (e.g., a comment that consisted solely of the word “first” so as to indicate that the commenter was the first to comment on the video in question), and regional dialects/differences complicated matters, particularly as many of the coders were located in different countries and had different native languages. Coders agreed less than 50% of the time on which codes to assign, although most pairs of coders were in agreement on which *categories* to assign approximately 70% of the time. Kappa values for each pair of coders ranged from .3 to.4. The two coders with the highest rate of agreement discussed the scheme via e-mail and Skype; two further rounds of coding were required before a satisfactory Kappa value had been produced (in this case, .63). Although the comments used for codebook testing had been drawn from the 5854 sampled comments, it was decided to recode all of these comments once a satisfactory level of agreement had been reached. Each of the two coders was responsible for roughly one half of the sample.

## Results

### Stage 1

Table 4 reports the results of the content analysis after the arbitration process, together with tests of significance that measure the relationship between the codes assigned to videos posted on YouTube and the codes assigned to videos posted on TED. The reported percentages represent the percent of comments with each type of interaction; as multiple categories could be assigned to each comment, the total exceeds 100%. Results are the values of the two main coders when they agreed and the values after arbitration by another coder when they disagreed. A significantly greater proportion of the sampled TED website comments (72.7%) engaged with the talk content than the proportion of YouTube comments (56.7%), although the main source of this difference is the summarizing of key points from the talk (2b) rather than a more critical analysis (e.g., 2e). The platforms were significantly different in the degree to which they encouraged interaction: YouTube comments were statistically more likely to engage in discussion with previous commenters (24%) than TED comments (12.3%). Personal insults were significantly more prevalent on the YouTube platform (5.7%) than the TED platform (less than 1%).

**Table 4:** A comparison of the broad types of comments between the two sites.

Type of interaction	TED site	YouTube	P value
1. Comment on speaker OR talk style BUT NOT relating to talk content	16.0%	15.0%	0.7350
*1a Personal anecdote (self-identification with speaker)*	0.7%	0.0%	0.1466
*1b Criticism of speaker (not the talk or message)*	1.0%	4.0%	0.0186
*1c Praise of speaker (not the talk or video)*	12.7%	4.7%	0.0005*
*1d Comment on speaker demographics*	1.0%	3.7%	0.0290
*1e Other comment on speaker*	0.7%	2.0%	0.1677
*1f Comment on speaker delivery/style (with or without praise or criticism)*	1.7%	2.3%	0.5997
2. Comment on talk content	72.7%	56.7%	<0.0001**
*2a Personal anecdote relating to talk content*	6.7%	3.7%	0.0980
*2b Summarize talk or reiterate key point from talk*	11.0%	2.7%	<0.0001**
*2c Praise of talk content (without any discussion of talk)*	14.3%	10.3%	0.1358
*2d Criticism of talk content (without any discussion of talk)*	1.0%	3.0%	0.0802
*2e Discuss issue related to talk*	21.3%	18.7%	0.4260
*2f Discuss talk - agreement/praise* *(discuss means make a point about the talk)*	11.0%	6.0%	0.0281
*2g Discuss talk - disagreement/criticism*	7.7%	8.7%	0.6553
*2h Discuss talk - other (without praise or criticism); includes* *neutral questions & speculations OR simple pointers* *to information*	12.7%	10.7%	0.4460
3. Other interaction with previous commenter withNO discussion of talk content	12.3%	24.0%	0.0002**
*3a Insult previous commenter*	0.7%	5.7%	0.0005*
*3b Praise previous commenter*	0.7%	0.0%	0.1466
*3c Agree with previous comment without discussion*	1.7%	2.7%	0.4037
*3d Disagree with previous comment without discussion*	2.3%	3.7%	0.3148
*3e Any other interaction with previous commenter without any discussion of talk content*	7.7%	13.7%	0.0174
4. Meta comment about TED itself	6.0%	3.0%	0.0763
5. Spam (includes self-promotion unrelated to talk)	0.0%	1.7%	0.0233
6. Self-promotion	1.0%	0.3%	0.2860
Contains pointer to external information	9.0%	7.7%	0.5649

+p values are from differences in proportions tests. Bonferroni corrections for 26 simultaneous tests lower 0.05 to 0.001,923, 0.01 to 0.000,385 and 0.001 to 0.000,039.

*Sig. at p = 0.05,

**sig. at p = 0.01,

***sig. at p = 0.001.

These results suggest statistically significant differences in the utility of the two platforms and the way in which they facilitate or hinder certain types of communication. Therefore, the next stage of the project sought to identify whether differences were also exhibited based on presenter characteristics.

### Stage 2

As with stage 1, difference between proportions tests were used to analyze each of the variables independently and in pairs. Table 5. addresses differences in comments by platform; please note that this stage drew upon a *different* set of videos. Whereas stage 1 was limited to videos tagged by TED as “science” or “technology,” stage 2 considered *all* videos and then sampled out presenters based on the lowest common denominator (in this case, female academics).

**Table 5:** Difference in proportions of comments of various types between platforms.

	YouTube %	TED %	Sig. p+
Comment on Speaker	9.8%	15.2%	0.000,000***
1P	4.1%	11.6%	0.000,000***
1N	3.9%	2.1%	<0.0001**
1U	1.4%	0.9%	0.0335
1M	0.4%	0.7%	0.1636
Comment on talk	60.8%	85.3%	0.000,000***
2P	24.4%	45.0%	0.000,000***
2N	12.4%	8.3%	0.000,000***
2U	21.3%	26.1%	0.000,023***
2M	2.7%	6.0%	0.000,000***
Interaction with commenter	32.8%	27.9%	<0.0001**
3P	5.5%	8.0%	<0.0001**
3N	14.4%	6.8%	0.000,000***
3U	12.2%	11.6%	0.4652
3M	0.7%	1.5%	0.002,452
About TED	4.0%	4.0%	0.9972
4P	1.5%	2.0%	0.1001
4N	1.7%	0.9%	0.009,905
4U	0.8%	0.9%	0.4927
4M	0.1%	0.2%	0.7488
Other	9.8%	1.3%	0.000,000***
5P	0.2%	0.0%	0.1000
5N	0.3%	0.1%	0.1276
5U	9.3%	1.1%	0.000,000***
5M	0.0%	0.0%	0.3194

+p values are from differences in proportions tests. Bonferroni corrections for 25 simultaneous tests lower 0.05 to 0.002, 0.01 to 0.000,4 and 0.001 to 0.000,04.

*Sig. at p = 0.05,

**sig. at p = 0.01,

***sig. at p = 0.001.

TED tended to provoke more discussion about the speaker or talk content, whereas YouTube tended to encourage interaction between commenters. In all three cases, TED received more positive codes than YouTube; this was significant when commenters were discussing the speaker or the talk, or if commenters were interacting with each other. Due to a large number of spam cases, YouTube had a disproportionate number of “5U” comments (e.g., YouTube comments often tend to self-congratulate by being the first to respond by stating comments such as “First,” “Second,” etc.). These findings largely reinforce what was found in Stage 1, emphasizing the significant differences in commenting between platforms.

Differences in comments according to the presenter’s gender are shown in Table 6. In terms of the high level categories, there were no differences in the degree to which commenters discussed the talk, interacted with each other, spoke about TED, or made irrelevant comments. However, there was a significant difference in the manner in which the presenter’s style or appearance was discussed. That is, commenters were more likely to discuss the presenter if she was female. Furthermore, there were significant differences in the sentiment of the comments when the speaker was discussed: comments tended to be more emotional when discussing a female presenter (significantly more positive *and* negative). Conversely, comments about the speaker tended to be more neutral when the presenter was male, although this was not on the level of statistical significance.

**Table 6:** Differences in comments by presenter’s gender.

	Female	Male	Sig. p+
Comment on speaker	15.28%	9.84%	0.000,000***
Positive	9.87%	5.89%	0.000,000***
Negative	3.80%	2.18%	<0.0001**
Neutral	0.82%	1.46%	0.01773
Mixed	0.79%	0.31%	0.01403
Comment on talk	73.03%	73.23%	0.2620
Positive	35.23%	34.23%	0.7013
Negative	10.52%	10.12%	0.7532
Neutral	22.76%	24.66%	0.04202
Mixed	4.52%	4.22%	0.6609
Interaction with commenter	30.60%	30.01%	0.9109
Positive	7.75%	5.76%	0.003995
Negative	10.86%	10.32%	0.6329
Neutral	11.03%	12.77%	0.02358
Mixed	0.96%	1.16%	0.4230
About TED	4.35%	3.75%	0.2954
Positive	1.71%	1.81%	0.7311
Negative	1.64%	0.95%	0.02319
Neutral	0.79%	0.89%	0.6444
Mixed	0.21%	0.10%	0.3234
Other	5.79%	5.21%	0.4039
Positive	0.07%	0.14%	0.4078
Negative	0.24%	0.14%	0.3731
Neutral	5.45%	4.94%	0.4562
Mixed	0.03%	0.00%	0.3194

+p values are from differences in proportions tests. Bonferroni corrections for 25 simultaneous tests lower 0.05 to 0.002, 0.01 to 0.000,4 and 0.001 to 0.000,04.

*Sig. at p = 0.05,

**sig. at p = 0.01,

***sig. at p = 0.001.

The provenance of these emotional comments can be seen in Table 7. As shown, there was very little distinction between positive and negative comments about male or female speakers on the YouTube platform, in the sense that commenters were equally *emotional* (either positive or negative) depending on the gender of the presenter. There was a larger range between positive and negative comments on the TED platform, which tended to be more positive on the whole, particularly in regard to women.

**Table 7:** Differences in types of comment by platform and gender.

	YouTube female	YouTube male	TED female	TED male
Comment on speaker	12.8%	6.9%	17.7%	12.8%
Positive	5.9%	2.3%	13.7%	9.5%
Negative	5.2%	2.6%	2.4%	1.8%
Neutral	1.2%	1.7%	0.5%	1.2%
Mixed	0.6%	0.3%	1.0%	0.3%
Comment on talk	59.8%	61.9%	86.1%	84.6%
Positive	23.6%	25.2%	46.7%	43.3%
Negative	13.1%	11.7%	8.0%	8.5%
Neutral	20.2%	22.4%	25.2%	26.9%
Mixed	2.9%	2.5%	6.1%	5.9%

Differences in commenting behavior according to the presenter’s academic status are examined in Table 8. These results are fairly similar to the analysis between men and women in that the only significant difference in high level categories is for the degree to which the speaker is discussed, with the non-academic speakers discussed more than the academics. In terms of sentiments, commenters were significantly more positive when discussing non-academic speakers and talks and more neutral when discussing academic talks.

**Table 8:** Differences in comments by academic status.

	Academic	Non-academic	Sig. p+
Comment on speaker	10.51%	14.62%	<0.0001***
Positive	5.75%	10.02%	0.000,000***
Negative	3.33%	2.64%	0.1210
Neutral	1.05%	1.24%	0.4944
Mixed	0.37%	0.72%	0.06877
Comment on talk	72.35%	73.92%	0.1754
Positive	32.72%	36.75%	0.001203*
Negative	9.83%	10.81%	0.2178
Neutral	25.75%	21.65%	0.000,226**
Mixed	4.05%	4.70%	0.2240
Interaction with commenter	31.70%	28.89%	0.01933
Positive	7.14%	6.35%	0.2283
Negative	10.68%	10.50%	0.8229
Neutral	13.06%	10.74%	0.006,134
Mixed	0.82%	1.30%	0.072,82
About TED	3.98%	4.12%	0.7859
Positive	1.87%	1.65%	0.5222
Negative	1.22%	1.37%	0.6117
Neutral	0.75%	0.93%	0.4504
Mixed	0.14%	0.17%	0.7704
Other	5.92%	5.08%	0.1587
Positive	0.07%	0.14%	0.4080
Negative	0.14%	0.24%	0.3794
Neutral	5.71%	4.67%	0.07295
Mixed	0.00%	0.03%	0.3476

+p values are from differences in proportions tests. Bonferroni corrections for 25 simultaneous tests lower 0.05 to 0.002, 0.01 to 0.000,4 and 0.001 to 0.000,04.

*Sig. at p = 0.05,

**sig. at p = 0.01,

***sig. at p = 0.001.

These findings suggest that differences in comments by presenter demographics are mainly found in response to discussions about the presenter, rather than the content of the talk or discussion amongst commenters. The tendency of commenters to discuss the presenter’s characteristics when the speaker was a non-academic may reflect the fact that many non-academic presenters were musicians or other celebrities, for whom visual appeal and stage presence is a particularly critical concern. In addition, the presumably scholarly nature of academics’ talks may be the reason why comments on such videos tended to be focused on neutral discussions of talk content (as opposed to *emotional* discussions of the talk content).

## Discussion

Stage 1 of the analysis demonstrated that there were significant differences between platforms in regard to the manner in which commenters interacted with the videos in question. Specifically, people were more likely to interact with the talk content on the TED site, particularly in terms of summarizing the talk or reiterating key points from the presentation. Conversely, people were more likely to interact with other commenters on the YouTube website, and a significant number of these interactions were negative. It should be noted that these comments did *not* discuss the talk content, even peripherally. There are some limitations in regard to the content analysis results in stage 1. From a sampling perspective, the comments were randomly selected according to unique videos; a random selection of *comments* would require a complete list of comments for all of the relevant videos. Accordingly, the results reflect the average per presenter rather than the average per comment. The subjectivity of the human coding element is another limitation. Although a fair to good level of inter-coder agreement was obtained for the major categories, the minor category results are less reliable, despite the arbitration used. In addition, the coders frequently had to interpret comments out of their original context, and thus the intentions of such comments may have been misunderstood.

Stage 2 of the analysis revised the coding scheme used in Stage 1. Several rounds of coding were required in order to clarify the sentiments and categories that were to be assigned to comments that were sarcastic, ambiguous, etc., and the very nature of textual discourse may have meant that some sentiments were misinterpreted or overlooked entirely. In this stage of coding, a substantial proportion of the comments left on YouTube (9.8%) were classified as “other/neutral,” which reflects the somewhat “spammy” nature of the YouTube site. By comparison, the comments section on the TED site was relatively “clean.” Note that in both YouTube and the TED website, users must register with the site in order to post a comment. This seems more likely to introduce a commenter/viewer bias in the TED website since a person would have to register specifically for commenting on a TED video. In contrast, YouTube viewers might have previously registered with YouTube to comment on other videos or to upload their own videos. This is particularly interesting to consider in light of the finding that comments on the TED website tend to be more positive than the comments left on the YouTube site. One possible interpretation is that people who go to the TED website in order to view videos are already invested in the TED philosophy (and thus receptive to the themes, talks, and presenters evidenced in the videos), whereas YouTube viewers can “stumble upon” a talk without any previous knowledge of (or affection towards) TED. This might also partly explain why there are more neutral comments about talks on TED than on YouTube; as seen in stage 1, commenters on the TED website engage with the talk content on a deeper level than simply agreeing or disagreeing with the presenter’s views.

The findings from stage 1 and 2 answer the first research question in the affirmative: platform matters. Although commenters are more likely to engage with talk content on the TED website than they are on the YouTube website, a majority of comments on YouTube still related to the ideas present in any given video. In addition, whilst the results may not completely allay the fears of those who worry that TED Talks give a misleading impression of science, perhaps Taleb’s idea of TED Talks being “a monstrosity that turns scientists and thinkers into low-level entertainers” [22] can be finally called into question.

The second research question sought to understand the relationships between presenter characteristics and comments. The results demonstrated that gender and academic status of the presenter both had significant effects in the degree to which comments discussed the presenter–but non-significant differences in the degree to which commenters discussed the talk or engaged in conversations with each other. Previously, Sugimoto and Thelwall found that academic presenters received a significantly higher proportion of YouTube Likes (to dislikes) than non-academic presenters [16]. However, we found that there were more positive sentiments towards *non*-academic speakers (both in terms of their appearance/presentation style and the presentations themselves). This may be indicative of a viewing audience that is more warmly receptive to musicians and entertainers than it is to more scholarly discourse. This is reinforced by the sentiment expressed in regards to non-academic presenters: commenters were more likely to express positive and negative comments in regards to non-academics as compared with academic presenters. A similar finding was found in regards to female presenters: Commenters tended to be more “emotional” when the presenter was a woman; specifically, comments about the presenter were more likely to be positive *or* negative.

Ultimately, the results demonstrate that the majority of comments (regardless of platform) are engaging with the talk topic in some fashion, perhaps reinforcing the notion that this dissemination vehicle is providing a platform for individuals to engage with and discuss ideas that range from scientific theories to magic tricks. A community of people interested in discussing “ideas worth spreading” has gathered on the two platforms, and this community engages with science and thoughts to a substantial degree, even if it is not committed to them exclusively. However, this is not a completely equitable space–the types of discourse vary significantly by platform and by presenter characteristic. It should be noted that this does not dramatically change how commenters respond to the talk; rather, it affects the manner in which they respond to the *presenter*.

## Future Research

Contemporary researchers have available to them a plethora of publicly available, naturally-occurring data sources. These datasets have the potential to transform scholarly research and enhance the public good [35], particularly in regard to social systems [36] and societal problems [37]. Analysis of online trends and activities can reveal insights into consumer behaviors [38], forecast financial patterns [39]–[41], detect the outbreak of medical epidemics [42], and even demonstrate connections between a country’s GDP and the degree to which its citizens use Google to locate information about the future (as opposed to the past) [43]. Researchers can now address questions that were previously impossible to answer, and our research can be seen as one of many possible ways to make use of these publicly available datasets in order to answer questions across a wide range of topics.

While the current method of analysis was unobtrusive, it was also rather limited, given that it only considered those people who *commented* on a video. While it is difficult to envision a practical solution to this particular form of self-reporting bias, it would be instructive if a future study were able to sample from *all* viewers (perhaps by including a survey link on the relevant websites; while this would not eliminate a response bias, it would mitigate its effects). This would allow researchers to gain different insights into the behavior and attitudes of those individuals who consume TED videos, particularly as one would presume that individuals who decide to leave comments would tend to be more engaged with the talk than those who did *not* comment. That having been said, analyzing comments is logical because these are presumably left by people immediately after viewing a video (a documentation advantage that is rare for social research).

Other studies could investigate viewers’ depths of engagement with the talks (as opposed to the nature of their engagement), as well as conducting cross-analyses that take into consideration other characteristics of the presenters or their videos (e.g., if the video can be classified as “entertainment” in the form of a musical performance or magic act, the age of the presenter, the length of the talk, etc.). Finally, although gender was a key element of this study, the genders of the *commenters* was not known. YouTube is known to be predominantly male-dominated, but no similar statistics are available for the TED website, nor is it known if the audience for TED videos on YouTube differs substantially from the general YouTube population. At the present moment it is difficult to determine the gender of a commenter, given the preference for aliases (as opposed to, say, using “John Smith” as one’s username) on both sites. However, a study that was able to ascertain commenter gender (or other demographic characteristics) would allow for a more robust analysis and would provide further insights into the nature of the “community of curious souls” that has gathered around the TED initiative.
